# Both maternal and paternal risk factors for term singleton low birthweight infants in rural Chinese population: a population-based, retrospective cohort study

**DOI:** 10.1038/s41598-018-30036-1

**Published:** 2018-08-22

**Authors:** Shi Chen, Yingying Yang, Yimin Qu, Yun Zou, Huijuan Zhu, Hongbo Yang, Fengying Gong, Linjie Wang, Yu Jiang, Bill Q. Lian, Cynthia Liu, Chengsheng Yan, Jianqiang Li, Qing Wang, Shi-kun Zhang, Hui Pan

**Affiliations:** 1 Key Laboratory of Endocrinology of National Health and Family Planning Commission, Department of Endocrinology, Peking Union Medical College Hospital, Chinese Academy of Medical Science and Peking Union Medical College, Beijing, 100730 China; 2 The second hospital of Jilin University, Changchun, 130014 China; 3 School of public health, PUMC, Beijing, 100730 China; 4 University of Massachusetts Medical Center, 55 Lake Ave. North, Worcester, MA 01655 UK; 5 University of Kansas School of Medicine, 3901 Rainbow Blvd, Kansas City, KU 66103 UK; 6 Hebei Center for women and children’s health, Shijiazhuang, 050031 China; 7 School of Software Engineering, Beijing University of Technology Beijing, Beijing, 100124 China; 8 Tsinghua National Laboratory for Info. Science and Technology, Tsinghua University, Beijing, 100084 China; 9 Research association for women and children’s health, Beijing, 100081 China

## Abstract

No large population-based study has focused on both maternal paternal risk factors for low birthweight (LBW) in China. We aimed to identify parental risk factors associated with LBW.A population-based, retrospective cohort study was conducted on 202,725 singleton infants at 37–42 weeks. These term singleton newborns were classified as LBW with birthweight ≤2500 g(TLBW) and normal birthweight between 50^th^ to 97^th^ percentile (TNBW 50^th^–97^th^) according to Chinese singleton norms. Multiple logistic regression analyses were used to find those parental risk factors of LBW by comparing two groups. TLBW and TNBW(50^th^–97^th^) occupied 4.8% and 70.8% of the study population, respectively. Logistic regression showed a significant association with positive maternal hepatitis B surface antigen (RR = 1.979, P = 0.047), irregular folic acid intake (RR = 1.152, P = 0.003), paternal history of varicocele (RR = 2.404, P = 0.003) and female babies (RR = 1.072, P = 0.046). Maternal smoking, hypertension and history of stillbirth were found related to LBW but no statistically significant. Positive maternal hepatitis B surface antigen, irregular folic acid intake, paternal history of varicocele had a negative effect on birth weight. Measures are necessarily taken to avoid them to improve pregnancy outcomes. Further studies should be done to investigate each detailed risk factors on LBW.

## Introduction

Birth weight is a convenient factor indicating adverse outcomes of infants. Low birthweight infant has a higher perinatal mortality and it also increases health risk in adulthood^1^. The definition of low birthweight(LBW) infant was recommended as infant ≤2500 g regardless of gestational age by The World Health Organization(WHO) in 1950^2^. The incidence of LBW was 6.1% in mainland China reported by Chen Y *et al*.^3^ and 8.1% in Foshan of China by Rao J *et al*.^4^. The risk factors of LBW become important in lowering the incidence of LBW. With the support of National Health and Family Planning Commission of China(NPCP), our study covered 60% pilot counties with over 200,000 population in total. Although several studies concluded some common maternal risk factors associated with LBW, few studies included paternal factors. Some experts started to suggest the consideration of paternal information in studies aimed at the risk factors model of LBW. We are the first large study to analyze both maternal and paternal risk factors of LBW in China.

## Results

### Prevalence of term low birthweight infants

**Figure 1:**
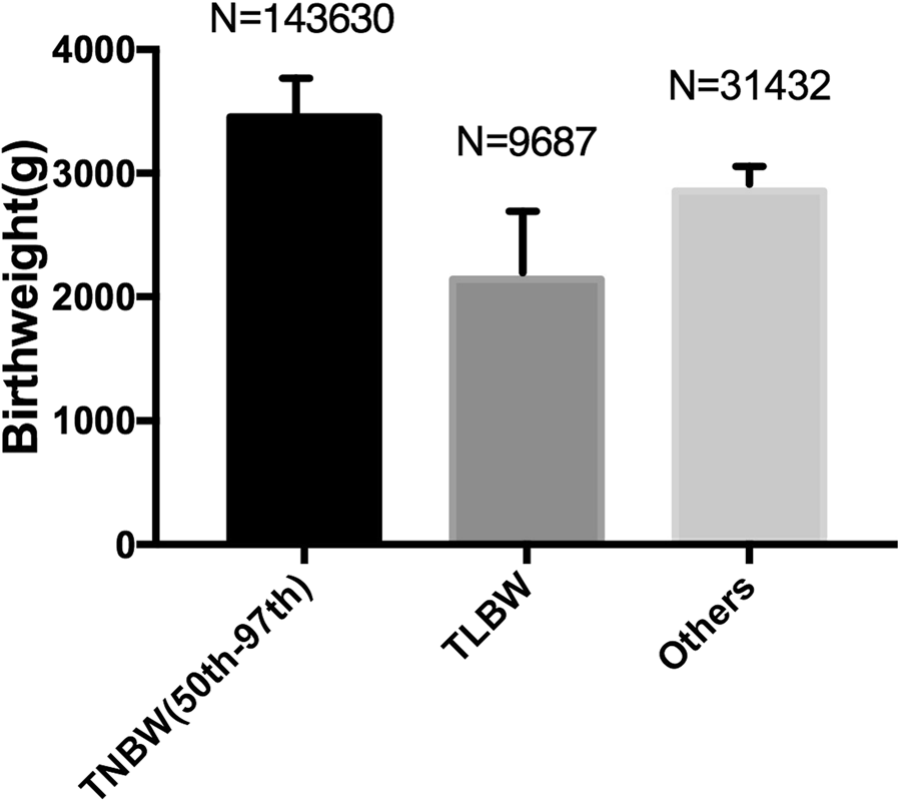
The distribution of term babies among different birthweight groups and comparison of mean birthweight of 3 groups. TNBW = Term normal birthweight; TLBW = Term low birth weight; Tmacrosomia = Term macrosomia; Others included birthweight of infants between 2500 g to 50^th^.

202,725 cases were analyzed in our study, the prevalence of term low birthweight infants(TLBW) was 4.8% (n = 9687) in rural China with the mean birthweight was 2140.54 ± 402.53 g. Among term low birthweight babies, 647 of them were very low birthweight infants. Term infants between 50^th^–97^th^ (TNBW 50^th^–97^th^) accounted for 70.8%(n = 143,630) and it had the highest mean birthweight (3454.34 ± 297.76 g). The remaining term babies were 31432 with the mean birthweight was 2855 ± 147.66 g. Figure 1. showed the distribution and compared the mean birthweight of these 3 groups.

### Univariate analysis

**Figure 2:**
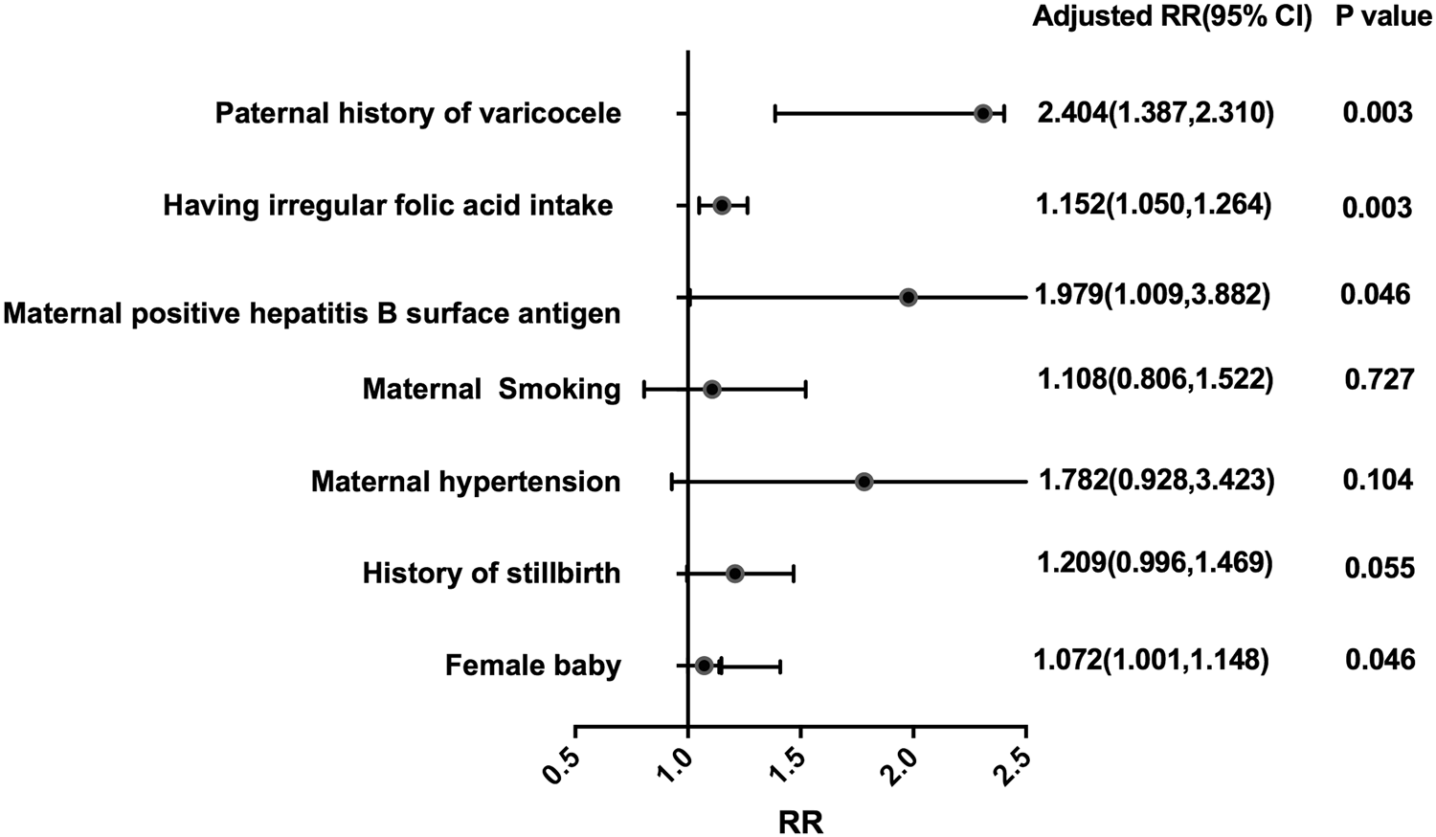
Independent risk factors found in multiple logistic regression analysis. RR = Relative risk. Cl = Confidence interval. The vertical bar represented RR = 1 and horizontal lines represented the range of adjusted RR of each risk factors. The reference group were: No history of varicocele, having regular folic acid intake, negative hepatitis B surface antigen, maternal non-smoking, no history of stillbirth, baby sex = male; RR were adjusted by maternal age and educational level.

In this study, maternal weight, height, BMI, age, hypertension, history of stillbirth, contraception, irregular folic acid intake, white blood cell count(WBC), red blood cell count, positive hepatitis B surface antigen, gestational age and paternal weight, height, BMI, age, history of varicocele and female infant were found significant in univariate analysis. Table 1. compared TLBW and TNBW (50^th^–97^th^) group, maternal hypertension and smoking in our study had a negative effect on birthweight of infants. However, it was no statistically significant. Maternal positive hepatitis B surface antigen was associated with decreased birthweight in our study (RR = 1.979, P = 0.046). Birthweight in regular folic acid group was higher than irregular group (RR = 1.152, P = 0.003). Additionally, Paternal history of varicocele would significantly increase the risk of LBW (RR = 2.404, P = 0.003). Female babies had a little higher risk of low birthweight (RR = 1.072, P = 0.046) (Fig. 2).

**Table 1:** The distribution of LBW and NBW(50^th^–97^th^) in univariate analysis.

	NBW(50^th^–97^th^)	LBW	T/χ^2^	P
Weight (♀)	53.49 ± 7.24	53.15 ± 7.55	4.843	<0.001
Height (♀)	159.25 ± 4.82	158.92 ± 4.86	7.425	<0.001
BMI (♀)			27.104	<0.001
<18.5	17682 (12.8%)	1327 (14.5%)		
18.5–24 (Ref.)	105304 (76.1%)	6774 (73.9%)		
24–28	12966 (9.4%)	890 (9.7%)		
28–32	1863 (1.3%)	136 (1.5%)		
≥32	555 (0.4%)	39 (0.4%)		
Age (♀)	25.13 ± 3.88	25.21 ± 3.90	−1.734	0.083
Age (♂)	27.23 ± 4.36	27.43 ± 4.42	−4.059	<0.001
Height (♂)	171.22 ± 5.14	170.89 ± 5.14	6.832	<0.001
Weight (♂)	65.74 ± 9.14	65.40 ± 9.20	3.938	<0.001
BMI groups (♀)			27.104	<0.001
BMI < 18.5	17682 (12.8%)	1327 (14.5%)		
BMI = 18.5–24 (Ref.)	105304 (76.1%)	6774 (73.9%)		
BMI = 24–28	12966 (9.4%)	890 (9.7%)		
BMI = 28–32	1863 (1.3%)	136 (1.5%)		
BMI ≥ 32	555 (0.4%)	39 (0.4%)		
Gestational age	39.25 ± 1.50	37.61 ± 4.12	46.378	<0.001
Maternal hypertension			1.264	0.261
No (Ref.)	133947(97.7%)	8847(97.5%)		
Yes	3222(2.3%)	230 (2.5%)		
History of stillbirth			54.995	<0.001
No(Ref.)	143567(99.9%)	9665(99.8%)		
Yes	63(0.1%)	22(0.2%)		
Maternal smoking			1.009	0.315
No (Ref.)	138276(99.7%)	9184(99.6%)		
Yes	483 (0.3%)	38(0.4%)		
Gum bleeding (♀)			1.082	0.298
No(Ref.)	131930 (95.5%)	8733 (95.3%)		
Yes	6204 (4.5%)	433 (4.7%)		
Folic acid intake (♀)			7.374	0.007
Regular (Ref.)	102962 (94.4%)	6797 (93.6%)		
Irregular	6113 (5.6%)	462 (6.4%)		
WBC (♀)	7.08 ± 3.30	7.18 ± 3.35	−3.097	0.002
Red blood cells (♀)			6.328	0.042
<3.5*10^12^/L	10548 (7.7%)	751 (8.1%)		
3.5–5.0*10^12^/L (Ref.)	118488 (86.2%)	7887 (85.3%)		
>5.0*10^12^/L	8351 (6.1%)	606 (6.6%)		
Hepatitis B surface antigen (♀)			8.451	0.015
Negative (Ref.)	130299 (95.4%)	8742 (94.8%)		
Positive	6263 (4.6%)	470 (5.1%)		
History of contraception (♀)			4.328	0.037
No (Ref.)	110280 (79.8%)	7238 (78.9%)		
Yes	27933 (20.2%)	1937 (21.1%)		
Paternal smoking			0.608	0.436
No (Ref.)	90377 (67.6%)	6010 (68.0%)		
Yes	43360 (32.4%)	2381 (32.0%)		
Paternal history of varicocele			13.683	0.000
No (Ref.)	143554(99.9%)	9673(99.9%)		
Yes	74(0.1%)	14(0.1%)		
Gender (infant)			8.726	0.003
Male (Ref.)	73367(51.1%)	4796(49.5%)		
Female	70263(48.9%)	4887(50.5%)		

Factors found significant by univariate analysis.”♂” means man, “♀” means woman. “Ref.” means “Reference group”.

## Discussion

LBW is associated with increased risk of diabetes mellitus, hypertension and cognitive dysfunction later during life^5^. But it is still not rare in developing countries with the overall prevalence was 15.9%^6^. To lower the incidence of LBW, it is very important to identify risk factors. Research have found maternal age, education level, BMI, ethnicity, socio-economic level, medical disease before and during pregnancy, health care and traffic air and noise pollution were related to LBW^6–8^. We found several important maternal and paternal risk factors which may involve in the process of LBW by a large, population-based database. Several studies have reported maternal hypertension and smoking were associated with LBW. Our current study showed the prevalence of LBW in maternal hypertension and smoking group were higher in those without hypertension and smoking before pregnancy but with no statistical significance in multiple risk analysis. Nevertheless, the number of rural smoked mothers were too small and many of them exposed to second-hand smoke which may mix the results. Interestingly, maternal exposure of hepatitis B surface antigen was found to be related to TLBW. Recent research also showed positive HBsAg may lead to a high risk of malformation and cesarean delivery but lower risk of macrosomia and non-significant higher risk of LBW in China^9^. The mechanism may be related to reduced blood supply of uteroplacenta due to the infection. This conclusion addressed the importance of management of maternal HBsAg carrier status. Several studies had demonstrated that folic acid supplementation was associated with decreased lowbirthweight infants. We found LBW easily happened in irregular folic acid intake group^10^. Maternal anemia is known to be related to folic acid supplementation, which will cause fetal hypoxia and intrauterine fetal distress^11^. Maternal anemia during pregnancy is an independent risk factor for low birthweight and preterm delivery. We are the first study found the relationship between low birthweight and paternal history of varicocele. Christman MS *et al*. tested the lower seman density and count in patients with a history of varicocele and cryptorchidism. We guess it is the main reason lead to LBW by zygotes with poor quality^12^. Further studies need to be done to address the mechanism. Our study also had limitations. Some of our data source were derived from questionnaire which lacked quantitative indices. Further, this study was conducted in rural area of China, so it was difficult to obtain the data covered whole China.

## Conclusion

Our study demonstrated that both maternal and paternal factors could affect birth weight. Maternal smoking and hypertension would lead to a non-significant LBW. Maternal positive hepatitis B surface antigen and irregular folic acid intake and paternal history of varicocele would increase the risk of LBW. In conclusion, more effort should be made to improve pre-pregnancy checkups to prevent LBW and long-term outcomes.

## Methods

### Description of our database system of NPCP

**Figure 3:**
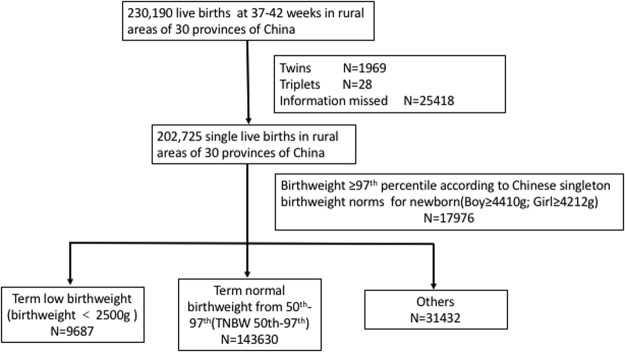
Study profile.

Our study was a population-based, retrospective cohort study including the information from database of National Pre-pregnancy Checkups Project (NPCP) organized by The Population and Family Planning Commission and The Ministry of Finance of China between January, 2010 and December, 2013. It covered rural areas of 30 provinces of China: Beijing, Hebei, Tianjin, Shandong, Zhejiang, Jiangsu, Anhui, Jilin, Fujian, Jiangxi, Henan, Hubei, Guangdong, Sichuan, Chongqing, Yunnan, Shanxi, Shanghai, Guangxi, Hunan, Hainan, Heilongjiang, Qinghai, Tibet, Inner Mongolia, Shanxi, Liaoning, Ningxia, Guizhou and Gansu. This project was built for couples planning on getting pregnant in rural China. Each couple will receive general information and medical history inquiry, physical examination, clinical laboratory examination and imaging examination. Included couples will be followed up on 12th weeks of gestation and 6 weeks after delivery. Last menstrual period was used to calculate the gestational age. Infants at 37–42 weeks were defined as term newborn. The group of NPCP has already worked on the risk factos of adverse pregnancy outcomes^13–15^. Our study included 248,501 records, among which ectopic pregnancy occurred in 176 cases and spontaneous abortion and self-induced abortion occurred in 5714 and 2526 pregnant women, respectively. 786 of records were stillbirths. 230,190 live births at 37–42 weeks were included, twins, triplets and those infants with missed birth information were first excluded. Full-term infants whose birthweight ≥97^th^ percentile according to Chinese birthweight percentile norm for newborn(Boy ≥4410 g; Girl ≥4212 g) were then excluded^16^. Finally, 202,725 single live births at 37–42 weeks were included in our study (Fig. 3).

### Data processing

The medical staffs from NPCP were trained by professional experts before collecting data and the database was built by application developers. To ensure the accuracy of data, questionnaires were completed by two trained interviewers at the same time. Our database had items including social demographics, illness and medical history, living habits, psychological status, environmental exposure, maternal pregnancy complications and biological parameters of parents (Table 2).

**Table 2:** Items included in our study.

Items	Contents
Social demographics	Pregnancy age, height of parents, nationality, education level, employment type, infants’ gender and body weight
Illness and medical history	Paternal history of hypertension, diabetes mellitus, thyroid disorders, nephritis, heart disease, tuberculosis, hepatitis
Parental living habits and nutritional status	Parental smoking and drinking status, maternal intake of folic acid and the time and length of folic acid use, Paternal use of contraceptives
Parental psychological status before and during pregnancy	Life or work pressure, tensions in relationships with their colleagues and relatives, preparedness for pregnancy
Environmental risk factors exposure	Parental exposure to radiation, organic solvents, pesticides and pets before and during pregnancy
Parental reproductive health	Maternal history of adnexitis, presence of bacterium, trichomonas, chlamydia trachomatis, neisseria gonorrhoeae infection in maternal vaginal fluid, evidence of parental treponema pallidum antibodies, maternal infection of cytomegalovirus IgM and toxoplasma IgM, paternal history of mumps, orchitis, epididymitis and varicocele
Maternal abnormalities during pregnancy	Vaginal bleeding, fever, diarrhea and abdominal pain during pregnancy
Parental biological parameters	Maternal systolic and diastolic blood pressure; hemoglobin, red blood cell, platelet, white blood cell count, neutrophil ratio, blood glucose, alanine aminotransferase, creatinine, thyrotrophic hormone and hepatitis B surface antigen; paternal alanine aminotransferase, creatinine, and hepatitis B surface antigen

Items included in our study. These items covered the maternal and paternal factors, including social demographics, living habits and nutrition, psychological status, environmental exposure risk factors, reproductive health, maternal abnormalities during pregnancy, and biological parameters, which were formulated by experts.

### Definition of variables and variable grouping

All included infants were divided into three groups: (1). Term low birthweight (TLBW): birthweight ≤2500 g; (2). Term normal birthweight from 50^th^–97^th^ (TNBW 50^th^–97^th^): Infants between 50^th^–97^th^ percentile with boy’s birthweight between 3073 g to 4410 g and girl’s birthweight between 2964 g to 4212 g. (3) The remaining infants. We included the following variable in our analysis including categorical variables and continuous variables. Table 3 showed the assignment of numeric variables.

**Table 3:** The assignment of numeric variables.

Variable	Group	Assignment
Blood pressure (BP)(♀)	Normal	0
	Systolic BP > 140 mmHg/diastolic BP > 90 mmHg	1
BMI (♀)	BMI < 18.5 Kg/m2	0
	BMI ≥ 18.5 Kg/m2 and BMI < 24 Kg/m2	1
	BMI ≥ 24 Kg/m2and BMI < 28Kg/m2	2
	BMI ≥ 28 Kg/m2 and BMI < 32 Kg/m2	3
	BMI ≥ 32 Kg/m2	4
Hemoglobin (♀)	110–150 g/L	0
	<110 g/L	1
	>150 g/L	2
Red blood cells (♀)	3.5–5.0*1012/L	0
	<3.5*1012/L	1
	>5.0*1012/L	2
Platelets (♀)	100–300*109/L	0
	<100*109/L	1
	>300*109/L	2
Leukocyte (♀)	4–10*109/L	0
	<4*109/L	1
	>10*109/L	2
Alanine (♀)	10–40 U/L	0
	<10 U/L	1
	>40 U/L	2
Creatinine (♀)	44–97μmol/L	0
	<44μmol/L	1
	>97μmol/L	2
Thyroid Stimulating Hormone (TSH) (♀)	2–10mU/L	0
	<2 mU/L	1
	>10 mU/L	2
Percentage of neutrophils (♀)	50–70%	0
	<50%	1
	>70%	2
Percentage of eosinophil granulocyte (♀)	0.5–5%	0
	<0.5%	1
	>5%	2
Percentage of lymphocyte (♀)	20–40%	0
	<20%	1
	>40%	2
Percentage of monocyte (♀)	3–8%	0
	<3%	1
	>8%	2

Variables assigned to different groups in our study. Table 2 shows how the variables were assigned to groups. In the table, “♂” means man and “♀” means woman.

### Ethical Approval

The informed consent was obtained from all subjects and all methods were carried out in accordance with relevant guidelines and regulations. All experimental protocols were approved by NPCP.

### Data Analysis

Continuous data were performed as the mean and standard deviation (SD) and categorical data were performed as frequency and percentage. The chi-squared test was used for qualitative data and independent sample t-test were used for quantitative data analysis. Variables with P value <0.1 was considered as significant in screening single factors. To address confounding variables, stratified analysis on all suspected confounders was used to estimate the specific risk for each stratum, stepwise multiple logistic regression was performed to correct the confounders in multi-factor analysis if the ajusted effect differs between each subgroup^17^. P < 0.05 was considered statistically significant. SPSS 24.0 edition was used for data analysis.
